# The Effects of Reduced Gluten Barley Diet on Humoral and Cell-Mediated Systemic Immune Responses of Gluten-Sensitive Rhesus Macaques

**DOI:** 10.3390/nu7031657

**Published:** 2015-03-06

**Authors:** Karol Sestak, Hazel Thwin, Jason Dufour, Pyone P. Aye, David X. Liu, Charles P. Moehs

**Affiliations:** 1 Division of Microbiology, Tulane National Primate Research Center, Covington, LA 70433, USA; E-Mail: hthwin@tulane.edu; 2 Division of Veterinary Resources, Tulane National Primate Research Center, Covington, LA 70433, USA; E-Mails: jdufour@tulane.edu (J.D.); paye@tulane.edu (P.P.A.); 3 Division of Comparative Pathology, Tulane National Primate Research Center, Covington, LA 70433, USA; E-Mail: dliu1@tulane.edu; 4 Arcadia Biosciences Inc., Seattle, WA 98119, USA; E-Mail: max.moehs@arcadiabio.com

**Keywords:** celiac, gluten, barley, gluten-free, NCGS, AGA, T cell, enteritis

## Abstract

Celiac disease (CD) affects approximately 1% of the general population while an estimated additional 6% suffers from a recently characterized, rapidly emerging, similar disease, referred to as non-celiac gluten sensitivity (NCGS). The only effective treatment of CD and NCGS requires removal of gluten sources from the diet. Since required adherence to a gluten-free diet (GFD) is difficult to accomplish, efforts to develop alternative treatments have been intensifying in recent years. In this study, the non-human primate model of CD/NCGS, e.g., gluten-sensitive rhesus macaque, was utilized with the objective to evaluate the treatment potential of reduced gluten cereals using a reduced gluten (RG; 1% of normal gluten) barley mutant as a model. Conventional and RG barleys were used for the formulation of experimental chows and fed to gluten-sensitive (GS) and control macaques to determine if RG barley causes a remission of dietary gluten-induced clinical and immune responses in GS macaques. The impacts of the RG barley diet were compared with the impacts of the conventional barley-containing chow and the GFD. Although remission of the anti-gliadin antibody (AGA) serum responses and an improvement of clinical diarrhea were noted after switching the conventional to the RG barley diet, production of inflammatory cytokines, e.g., interferon-gamma (IFN-γ), tumor necrosis factor (TNF) and interleukin-8 (IL-8) by peripheral CD4+ T helper lymphocytes, persisted during the RG chow treatment and were partially abolished only upon re-administration of the GFD. It was concluded that the RG barley diet might be used for the partial improvement of gluten-induced disease but its therapeutic value still requires upgrading—by co-administration of additional treatments.

## 1. Introduction

CD is an autoimmune disease that affects approximately 3 million people in the United States (US) [1], although only a small fraction has been diagnosed. Furthermore, a non-autoimmune NCGS affects an estimated additional 6% of the population e.g., 20 million in the US [2,3]. Both CD and NCGS are characterized by sensitivity to dietary gluten. High prevalence of CD and NCGS in cereal grain-consuming societies highlights the need for novel treatments for gluten sensitivity and illustrates how many people may benefit from the successful outcome of related research.

A number of novel pharmacological strategies are currently being explored for the treatment of CD. These strategies include experimental drugs that reduce intestinal permeability, inhibitors of intestinal tissue transglutaminase (TG2), as well as major histocompatibility class II blockers [4]. Most of these therapies are still far from the clinic. An alternative therapy for CD and NCGS may include cereals whose storage proteins are modified to reduce the accumulation of the immunotoxic gluten epitopes [5]. The endosperm in cereals such as barley and wheat consists of 8%–14% protein; these storage proteins are prolamins and glutenins known colloquially as “gluten”. The major CD-eliciting epitopes have been found in the S-rich and S-poor prolamins [6,7,8]. Several groups have explored the natural variation present in wheat, barley and oats germplasm to determine if conventional plant-breeding approaches could be used to develop reduced gluten (RG) cereals [9,10,11,12,13]. Additionally, a transgenic approach to developing RG wheat was taken by Gil-Humanes and colleagues who down-regulated α, γ, and ω classes of wheat gliadins using the RNAi hairpin constructs of conserved gliadin sequences. The resulting transformed wheat had 10–20-fold lower content of immunotoxic epitopes [14,15,16].

Our group is focusing on a similar approach. Using a known RG barley mutant [17,18], the potential therapeutic benefits are evaluated in gluten-sensitive rhesus macaques. The barley mutant *lys3a* (RIS∅ 1508) was first identified in the early 1970s at an agricultural station in Denmark during the course of mutagenesis studies aimed at increasing the lysine content of barley, to enhance its nutritional value as animal feed [18]. This was successful; the lysine content was increased by 44% and follow-up experiments with rats and pigs confirmed superior nutritional properties of this mutant [19,20]. The increase in lysine in the *lys3a* mutant is due to a decrease in the accumulation of lysine-poor hordeins with a concomitant increase in the accumulation of more lysine-rich albumins and globulins [21]. These effects of the mutation resulted in a gluten content in the *lys3a* barley that is approximately 1% of that in the parental cultivar (Bomi). Here, we report the effects of conventional and RG barley-based primate diets (containing 10% by weight of Bomi or *lys3a* whole grain barley flour) in our gluten-sensitive rhesus macaque model.

## 2. Experimental Section

### 2.1. Ethics Approval

This study was performed with non-human primates. Ethics approval for veterinary procedures was obtained from the Tulane University Animal Care and Use Committee, Animal Welfare Assurance A-4499-01. All procedures were in accordance with the recommendations of the Guide to the Care and Use of Laboratory Animals (NIH) 78–23 (Revised, 1996).

### 2.2. Pre-Screening and Selection of Rhesus Macaques for the Study

The 200 young (1–3 years-old) rhesus macaques (*Macaca mulatta*) of Indian origin, belonging to Tulane National Primate Research Center breeding colony, were tested for the presence of serum anti-gliadin antibodies (AGA) as well as anti-TG2 antibodies as previously described [22] to identify suitable study subjects: Three AGA and TG2 antibody-negative macaques, without a clinical history of diarrheal illness (controls) and three AGA and TG2-positive macaques, with past histories of chronic diarrhea (gluten-sensitive e.g., GS) were assigned to the study. All six animals were Specific Pathogen-Free (SPF) e.g., negative for simian retrovirus type D, seronegative for simian T lymphotropic virus type 1, simian immunodeficiency virus and herpes B viruses, and free of selected enteric pathogens [23]. Tuberculin skin tests, performed semi-annually, were negative for each animal involved.

### 2.3. Diets Used

Upon assignment to the study animals were placed on a strict GFD, as described before [24], in order to accomplish immunological and clinical remission in GS macaques, characterized by baseline levels of AGA and TG2 serum antibodies and absence of diarrhea. The two barley-based diets were formulated in collaboration with Purina Inc., consistent with primate diet requirements. Conventional (variety Bomi) and RG barley (*lys3a*-derived from Bomi by mutagenesis) were obtained from the ARS National Small Grains Collection, Aberdeen, ID (http://www.ars.usda.gov/main/docs.htm?docid=2884). Both varieties were grown in southern California in the winter of 2013/2014 and harvested in the spring of 2014. The barley seeds were de-husked and milled to flour; gluten levels in the barley flours and the monkey chows were determined by Bia Diagnostics (http://www.biadiagnostics.com/) by the commercial immunoassay (R5 ELISA). Conventional and RG barley chows were manufactured by Purina Test-Diet. Barley flour was incorporated at 10% w/w of the chow. As measured by R5 ELISA, the conventional barley flour exhibited gluten levels between 175,000 and 240,000 parts per million (ppm) (mg/kg) while the RG barley flour ranged between 2000 and 3000 ppm. The gluten levels in conventional barley chow were approximately 16,000 ppm while in RG barley chow they were 200 ppm. Thus, both the RG barley flour and the corresponding chow contained approximately 1% of the gluten of conventional barley. Subsequently, gluten sources of traditional non-human primate chow (5K63) were replaced in Purina’s mixing facility with conventional barley flour (5BQF) and RG barley flour (5BQG). Respective chow pellets were color-coded brown and green. Based on amount of chow eaten daily (160–320 g of chow/day) and the level of gluten in the conventional and RG barley chow, we estimate that conventional barley chow delivered a dose of 2.5–5 g of gluten/day, while the RG barley chow led to an intake of approximately 32–64 mg of gluten/day.

### 2.4. Dietary Time Periods and Samples Collected

The six study macaques were kept on identical diets, during four periods, each period lasting approximately 2 months, in the following order: (1) GFD; (2) conventional barley-derived diet; (3) RG barley-derived diet and (4) GFD. Macaques were stationed in a Biosafety Level 2 (BSL2) facility, separated from the rest of the colony, to prevent contamination of their diet with gluten sources. When fed conventional barley chow, all six macaques had also access to a wheat bread. Five milliliters of EDTA blood and 1 g of stool were collected every two weeks from each animal to extract the serum and peripheral blood mononuclear cells (PBMC) as described [25] and to confirm the enteric pathogen-free status of studied animals [23]. Individual records of animal well-being and stool consistency (diarrhea) were recorded daily. Based on assessment of healthy control animals, diarrhea clinical scores were scaled in a range of 0–1, with values of >0.4 indicating diarrhea. To simplify and to summarize the scores, the animal group averages were calculated for every week.

### 2.5. Histopathological Evaluation, AGA, TG2 and Anti-Hordein Antibody (AHA) Serum Responses

A small intestinal biopsy sample (distal duodenum or proximal jejunum) was collected once every dietary period from each animal, as described [22,25]. The AGA and TG2 antibody-specific immunoassays were also performed [24,26]. In addition, an AHA immunoassay was used to determine if AGA responses overlapped with barley-induced AHA responses. Briefly, 10 mg/mL of pepsin-trypsin digested (PT)-gliadin was replaced with PT-hordein (Arcadia Bio.) as the coating antigen in AHA immunoassay to measure the AHA serum levels [26]. Histopathological evaluation of collected small intestinal biopsy samples was also done as previously described [24,26].

### 2.6. Fluorescent-Activated Cell Sorting (FACS) of Cytokine-Producing PBMCs

The FACS was performed according to standard protocols [27]. Briefly, PBMCs were stained with a mix of fluorescent antibodies from BD Pharmingen (San Diego, CA) unless otherwise noted: CD3 (SP34-2), CD4 (OKT4, Bio Legend, San Diego, CA), CD8 (SK1), CD20 (2H7, Bio Legend), CD152 e.g., CTLA-4 (BN13), TNF (MAb11), IFN-γ (4S.B3), IL-8 (G265-8, BD Horizon, San Jose, CA, USA) and IL-10 (JES3-9D7, Bio Legend). In order to detect the TNF, IFN-γ, IL-8 and IL-10 intracellular cytokines, lymphocytes were stimulated *in vitro* with 0.1 μM PMA and 0.5 μg/mL ionomycin (Sigma, St. Louis, MO, USA) and processed as described [27]. Samples were resuspended in BD Stabilizing Fixative (BD Biosciences, San Jose, CA, USA) and data acquired on FACSAria flow cytometer (BD Biosciences). Data were analyzed by the use of Flowjo software (Tree star, Ashland, OR, USA).

### 2.7. Statistical Analysis

The individual cytokine responses (proportions of parent peripheral lymphocytes secreting each of the pro- or anti-inflammatory cytokines) were compared between the control and GS groups of macaques by the use of Student T test. The probability *p < 0.05* was considered as significantly different.

## 3. Results

### 3.1. Serum Antibody Responses, Intestinal Histopathology and Diarrhea

**Figure 1:**
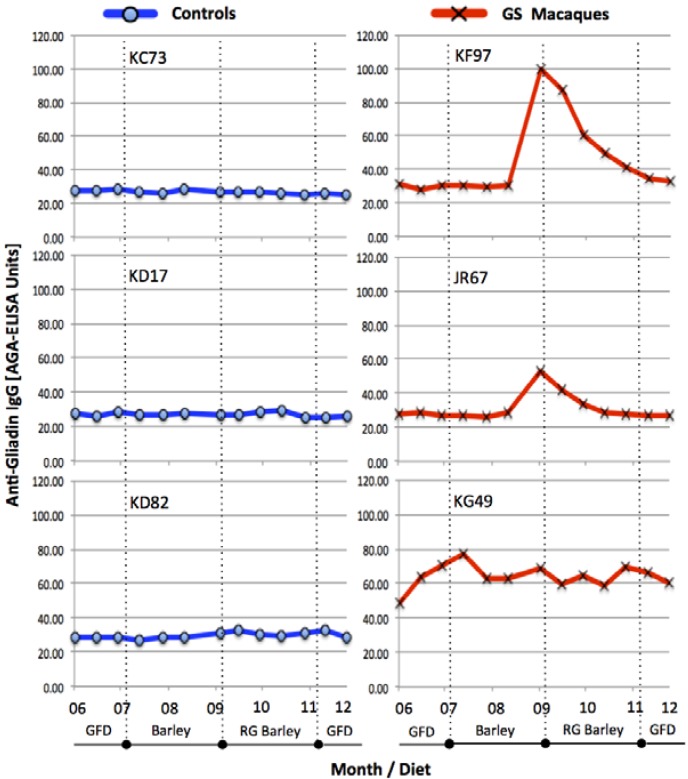
The kinetics of serum AGA antibody levels in three control (KC73, KD17 and KD82) and three GS (KF97, JR67 and KG49) macaques during the periods of (1) immunological remission e.g., GFD; (2) conventional barley diet; (3) RG barley diet and (4) GFD. Vertical, dotted lines indicate borders between different diets. Only the GS animals but not the healthy controls seroconverted to dietary gluten. The two GS macaques (KF97 and JR67) responded to its presence (AGA increase) as well as withdrawal (AGA decrease). KG49 macaque remained with elevated AGAs throughout the entire period of experiment regardless of dietary changes. AGA-ELISA cut-off = 40 Units.

In order to accomplish the immunological and clinical remission in GS macaques and to maintain the consistency between the diets of control and GS animals, all six macaques were first placed on a GFD. Two out of three GS animals (KF97 and JR67) responded well to the GFD and within one month decreased their AGA (as well as TG2, not shown) serum antibody levels to a base-range (Figure 1).

The third GS macaque (KG49) remained, despite the GFD treatment, with elevated AGAs suggesting that a longer GFD period might be required to remit AGAs of this animal. As anticipated, none of the healthy control macaques showed any AGAs before and during the study, regardless of dietary gluten intake (Figure 1). Upon introduction of conventional barley diet to GS macaques, KF97 and JR67, both animals responded with elevated AGAs while RG barley diet had the reverse effects (Figure 1). The AGA serum responses were followed by anti-hordein antibody (AHA) responses (not shown), confirming that measured serum antibody responses were, at least in part, induced by barley-derived prolamins.

**Figure 2:**
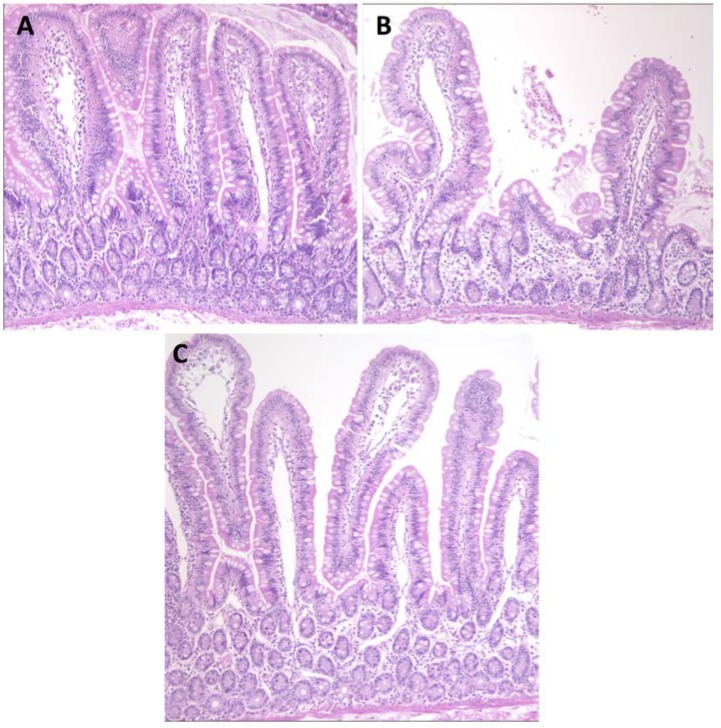
The effects of conventional and RG barley diets on small intestinal tissue architecture of control and GS macaques (H & E staining, jejunum, 100×). After being on conventional barley diet for 64 days, jejunum of control macaque (KD17) looks normal, unaffected, with only minimal lacteal dilatation (**A**) while GS macaque (KF97) jejunum is showing shortened villi e.g., villous atrophy (**B**). Multifocally, KF97’s lamina propria is moderately expanded by lymphoid aggregates. The lacteals are mildly dilated (lymphangiectasia). The submucosa is expanded by edema containing infiltrates of small number of neutrophils, eosinophils, lymphocytes and plasma cells (B). Follow-up treatment of KF97 with RG barley diet for 56 days resulted in an improvement: Jejunum became close to normal, with only moderate lymphangiectasia (**C**).

**Figure 3:**
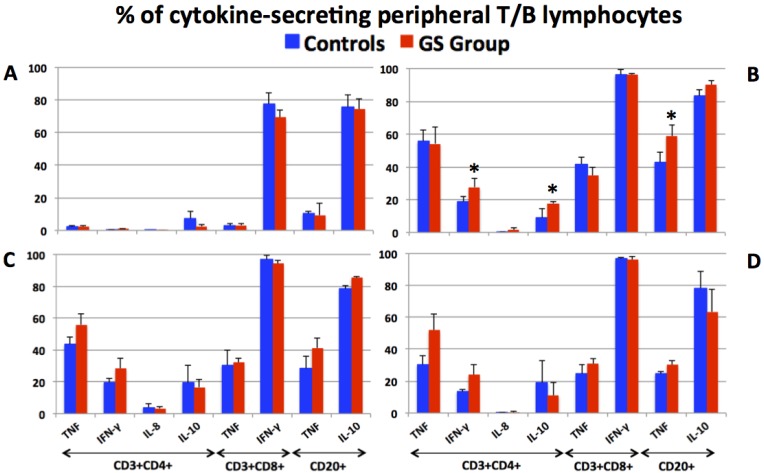
The production of inflammatory (IFN-γ, IL-8 and TNF) and anti-inflammatory (IL-10) cytokines by peripheral T helper (CD3+CD4+), cytotoxic T (CD3+CD8+) and B (CD3-CD20+) lymphocytes from control and GS macaques is shown at four selected time points. While only the minimal cytokine production by CD4+ T helper cells was measured at the stage of immunological remission induced by GFD (**A**), the use of conventional barley diet was characterized with significantly increased cytokine production: Day 64 is shown (**B**) with differentially increased cytokine production (*). An introduction of RG barley diet and its continuous administration for two months led only to transient decrease of TNF production by CD3+CD8+ T and CD20+ B cells in all animals (**C**). Upon reintroduction of GFD for one month, a further but still transient decrease of inflammatory cytokine-producing cells was noted (**D**) indicating the necessity of more sustained treatment.

**Figure 4:**
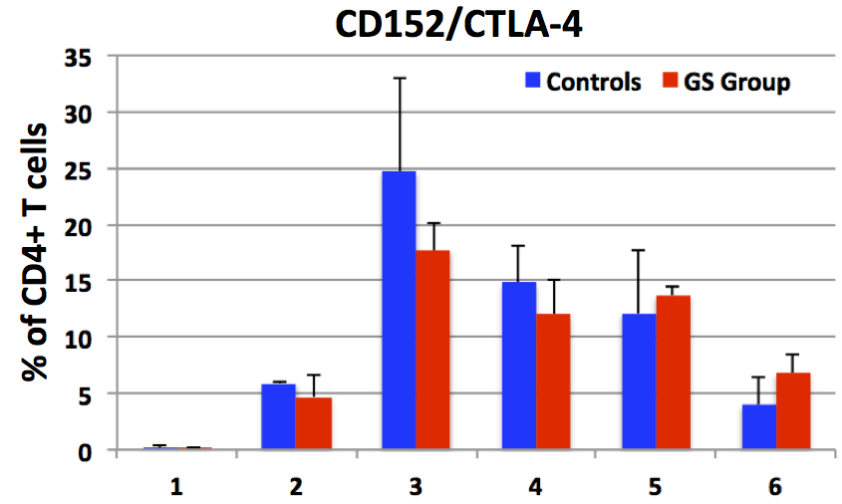
The proportions of CD152 (marker of T cell inhibition) expression by peripheral CD3+CD4+ T cells from control (C = blue columns) and gluten-sensitive (GS = red columns) macaques were evaluated. Six selected time points are shown including the GFD day 28 (X axis value 1); conventional barley diet days 14 (2) and 43 (3); RG barley diet days 28 (4) and 42 (5); followed again by GFD day 13 (6). While only the minimal expression of CD152 was measured during the first GFD period, an introduction of conventional barley diet led to an increased expression of CD152 by day 14, and further increase by day 43 in both groups. Replacement of conventional barley with RG barley diet did result in transiently lower % of CD3+CD4+CD152+ T cells. No significant differences in CD152 expression were observed between the two groups.

The AGA responses in GS macaques were paralleled with histopathological manifestations of gluten-sensitive enteropathy (Figure 2) as well as with clinical manifestations of diarrhea (Supplementary Figure S1). Although the effects of conventional barley diet were not as severe in GS macaques as previously described effects of wheat diet [24,25], both histopathological and clinical manifestations of villous atrophy and diarrhea were clearly present. Replacement of conventional barley with RG barley diet had beneficial effects not only on intestinal architecture (Figure 2C) but also for the improvement of clinical diarrhea scores (Supplement Figure S1). Despite these beneficial effects of RG barley diet, after two months on this diet mild enteritis and soft stools were still persisting in GS macaques. Such an incomplete remission of gluten-induced disease is in agreement with our past results when a wheat-free but not completely gluten-free diet was fed to GS macaques [24]. In this study, an incomplete remission of gluten-induced disease became more obvious when dissecting the expression of pro- and anti-inflammatory factors by peripheral CD4+ T helper cells (Figure 3 and Figure 4).

### 3.2. Production of Pro- and Anti-Inflammatory Cytokines by Peripheral T and B Lymphocytes

The populations of peripheral blood CD3+CD4+ T helper, CD3+CD8+ T and CD3-CD20+ B lymphocytes of control and GS macaques were identified (Supplementary Figure S2) and evaluated for the production of pro- and anti-inflammatory cytokines (Figure 3).

Selected time points representing the GFD, conventional barley, RG barley and GFD periods were included with the objective to illustrate the effects of RG barley diet on peripheral T and B cell cytokine responses. As anticipated, the baseline, associated with consumption of GFD, was characterized with very few cytokine-secreting cells of interest, namely the CD4+ T helper cells (Figure 3A). High proportions of CD8+IFN-γ+ T and CD20+IL-10+ B cells persisted throughout the experiment in both groups of macaques (Figure 3A–D), indicating that the presence of these cells was not due to dietary gluten changes. The two inflammatory cytokines that were affected by dietary gluten exposure were TNF and IFN-γ (Figure 3B–D). While there were no consistent differences between the control and GS groups with respect to TNF production, the IFN-γ-positive cells were consistently higher in the GS group. This difference became statistically significant by day 64 of the conventional barley diet (Figure 3B). Such a result indirectly corroborates the prominent role IFN-γ plays in pathogenesis of gluten-sensitive enteropathy e.g., GSE [28,29,30]. With the transition from the conventional barley to the RG barley diet, a transient decrease of TNF production by all three types of studied lymphocytes was observed regardless of group affiliation (Figure 3C). No further decrease of inflammatory cytokine-producing cells after feeding the RG barley diet continuously for two months (not shown) indicated that even the minute quantities of gluten contained in RG barley (200 ppm) were able to prevent remission of inflammatory cytokine production to baseline. Only when the animals were placed again on the GFD did cytokine production very slowly start returning to lower levels (Figure 3D).

### 3.3. Expression of CD152 by Peripheral CD4+ Cells e.g., Enumeration of CD4+CTLA-4+ T Helper Cells

CTLA-4 is normally expressed on the surface of CD4+ T helper cells from where it transmits immunoregulatory signals that in general suppress T cell mediated immune responses. Clinical significance of CTLA-4 agonists was implicated in the context of several autoimmune diseases including CD. In this study, the proportions of peripheral CD3+CD4+CD152+ T helper cells were assessed in GS and control macaques. It was hypothesized that increased numbers of these cells would indicate that GS hosts need to counter-balance the gluten-induced T cell responses. Thus, peripheral CD4+ T cell populations from control and GS macaques were evaluated for CD152 expression (Figure 4). While CD152 was only poorly expressed during the remission period (<1%), a robust increase of CD3+CD4+CD152+ cells was measured at days 14 and 64 of the conventional barley diet (Figure 4). Despite that a significant increase in CD152 expression was measured in both groups of macaques, no differences between the control and GS macaques were detected. With increasing time on the gluten-containing diet, the fraction of CD4+CD152+ to CD4+IFN-γ+ cells (anti- and pro-inflammatory T helper cells) started shifting in favor of IFN-γ cells in the GS macaque(s), while in the controls, this fraction was in favor of the CD4+CD152+ cells (Supplementary Figure S3). Reintroduction of the GFD had the expected effect—expression of both CD152 and IFN-γ dropped to levels closer to baseline (Figure 3 and Figure 4).

## 4. Discussion

This study was conducted with the objective to evaluate the treatment potential of RG barley-based diet. A GS rhesus macaque model was used to study the effects of RG diet, including humoral and T cell mediated immune responses. Consistent with past studies, experimental macaques were selected based on positive serum AGA and TG2 antibodies as well as clinical histories of chronic diarrhea of non-infectious etiology [22,25]. After being placed on a GFD for one month, both AGA and TG2 antibodies in two of the GS macaques dropped to baseline levels while the third GS animal was not responsive to GFD. This is in agreement with reports involving CD patients, according to which not all celiac patients respond promptly to GFD [31,32]. Consistent with our unpublished data, once the immunological remission is accomplished in GS rhesus macaques and dietary gluten is re-introduced, relapse of serum antibodies is faster in the case of AGA than TG2 auto-antibodies. This might be due to fundamentally different mechanisms of how AGA *vs.* TG2 antibodies are generated [31,33,34]. GS macaques chosen for this study were identified as both AGA and TG2 antibody-positive, however, since AGAs were more responsive to dietary gluten changes than the TG2 antibodies, AGA rather than TG2 antibody responses are presented in this manuscript to reflect the kinetics of the experimental diets.

Following the replacement of the GFD with the conventional barley diet, the two GS macaques that responded to dietary gluten withdrawal (KF97 and JR67), also responded to its re-introduction—with increased AGAs (Figure 1). These AGA responses were followed by serum anti-hordein antibody (AHA) responses (not shown), confirming that AGA responses were, at least in part, caused by barley-derived prolamins [35,36]. While AHAs were detected in serum from both control and GS animals, AGAs were only detected in GS macaques but not in controls. Seed storage proteins from related grasses including the gliadins from wheat and hordeins from barley share a number of proline and glutamine-rich epitopes; these are likely being recognized by both AGAs and AHAs [8]. Small intestinal histopathology and clinical diarrhea scores (Figure 2 and Supplementary Figure S1) corroborated the AGA results and provided additional clues: While all of the GS macaques showed evidence of GSE upon administration of the conventional barley diet, only partial remission of GSE was accomplished in these animals by the administration of the RG barley diet. This incomplete disease remission became even more apparent when comparing the numbers of pro- and anti-inflammatory peripheral blood lymphocytes at different time points (Figure 3 and Figure 4).

Because of their demonstrated and suspected involvement in CD and/or NCGS pathogenesis, the following factors were studied: IFN-γ, IL-10, IL-8, TNF and CD152/CTLA-4 [28,29,30,37,38,39,40,41,42,43,44,45]. The cells that were the subject of above analysis were peripheral lymphocytes including CD4+ T helper cells, CD8+ T cells and CD20+ B cells. It is important to underscore that all of the selected factors are known to play roles also in other inflammatory and autoimmune diseases [29]. Thus, to establish their order of significance in the GS macaques fed RG barley was the secondary objective of this study. In the case of human CD, a prominent role of IFN-γ was established due to its capability to activate the TG2 [30]. Therefore, an inhibition of the phosphatidylinositol-3-kinase pathway was suggested as a potential CD therapy [30]. Of the factors investigated in this study, the expression of pro-inflammatory IFN-γ by CD4+ T cells, followed by its IL-10 antagonist, reflected the dietary gluten changes more closely than the other studied factors. At several time points, the expression of IL-8, TNF and CTLA-4 were influenced by dietary gluten changes, however, without significant differences between the control and GS macaques. These results suggest that dietary gluten also affects to some extent healthy individuals. Interestingly and in contrast to AGA and cytokine responses, there was no significant trend for differences in CTLA-4 expression between the two groups of macaques. One consideration that could simplify observed effects in future studies is the use of genetically pre-selected macaques. In this study, GS subjects were selected by their TG2/AGA/clinical phenotype, without consideration of their *MHC II*, e.g., *Mamu II* characteristics. We recently showed that specific *Mamu II* alleles might predispose rhesus macaques to GS, analogous to the role played by human DQ2/8 alleles [25]. For the upcoming studies with novel CD therapeutic approaches, it would be beneficial to establish a genetically and phenotypically defined colony of CD and NCGS rhesus macaques, to initiate studies without the need to pre-screen large numbers of animals.

The results reported here demonstrate that the RG diet leads to a partial amelioration of gluten-induced symptoms in GS macaques. These results provide direction for the future use of RG barley. Despite its 100-fold lower content of dietary gluten, administration of RG barley still appears to trigger inflammatory responses in GS macaques.

The RG barley used, namely a mutant in the *lys3a* gene induced by a DNA alkylating agent, results in the simultaneous reduction of several classes of hordeins, including B and C hordeins. The molecular nature of the mutation is unknown, although it is hypothesized to be a lesion in a 5-methylcytosine DNA glycosylase enzyme that de-methylates DNA [46]. The altered DNA methylation of several hordein gene promoters in mutant endosperm compared to non-mutant parental endosperm has been found in the mutant [47]. An independent mutant, Riso 56, that contains a large deletion of B hordein genes [48], has been crossed with the *lys3a* mutant to create Ultra Low Gluten barley 2.0 (ULG), indicating that there is a potential to reduce barley gluten even further [49]. While ULG was reported to stimulate the *in vitro* reactivity of T cells 20-fold less than conventional barley [49], it has not yet been tested *in vivo*.

Other therapies that are being developed to treat CD such as glutenase enzymes with strong gluten degrading activity may be envisioned to synergize with a RG cereal to help break down the residual gluten [50,51,52,53]. Even if not yet appropriate for GS patients, the development of RG barley and other RG cereals may have additional benefits, such as increased content of the lysine in the grain. Future studies could also include morphometric end-points of macaques’ GSE, as described [24]. The future use of improved RG grains may ultimately contribute to a reduction in the incidence of gluten sensitivities.

## 5. Conclusions

This study was performed with the highly sensitive rhesus macaque model of gluten sensitivity to test the hypothesis that a RG barley mutant is less deleterious than its parental counterpart. Upon switching from the conventional to a RG barley diet, GS macaques exhibited an improvement in symptoms of gluten-induced disease, including GSE and diarrhea. Nevertheless, persisting production of inflammatory cytokines by peripheral lymphocytes indicated that complete remission could not be accomplished by the RG barley diet alone. Future studies should therefore focus on additional approaches to detoxifying the residual gluten.

## Supplementary Files

Supplementary File 1
