# Supplementary material for: The Effects of Reduced Gluten Barley Diet on Humoral and Cell-Mediated Systemic Immune Responses of Gluten-Sensitive Rhesus Macaques
Source: Nutrients. 2015 Mar 6;7(3):1657–71. doi: 10.3390/nu7031657 (PMC4377872; doi:10.3390/nu7031657)
Supplement: Supplementary File 1 [file nutrients-07-01657-s001.docx]

Supplementary Information


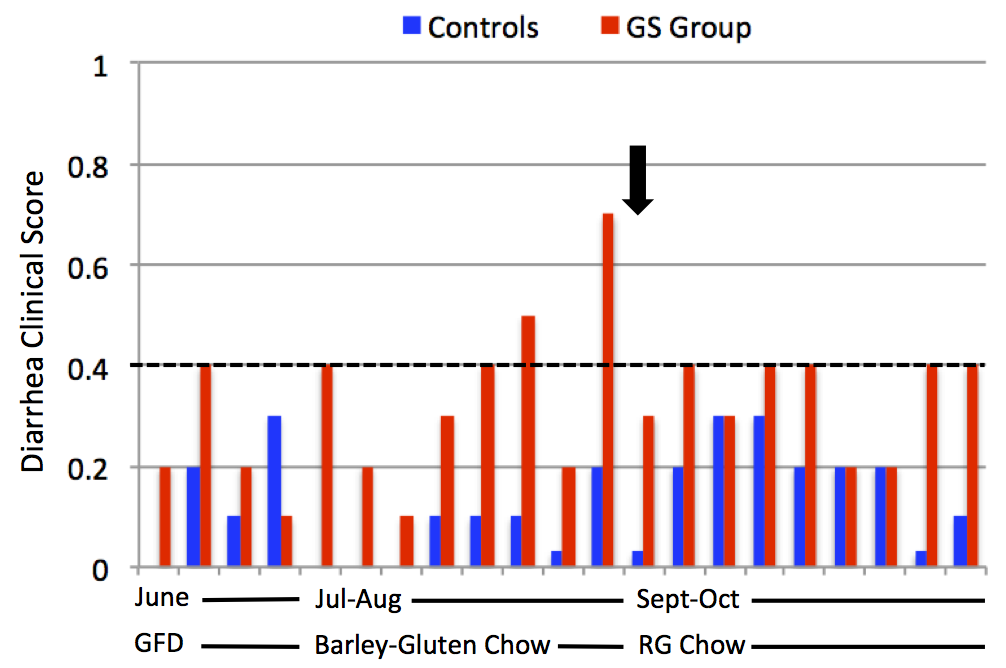


**Figure S1.** An average weekly stool/diarrhea clinical scores representing the control and GS groups of macaques during the three dietary periods: First period corresponds to GFD, second to barley chow (5BQF) and third to RG barley chow (5BQF). Arrow indicates the time when barley chow was replaced with RG chow. Stool clinical score of 0.4 indicates diarrhea baseline. Values above 0.4 indicate diarrhea, less than 0.4 normal stool while scores of 0.2–0.4 indicate some softer but still normal stools. During the second half of 5BQF diet period, only the GS but not the control macaques exhibited diarrhea scores of >0.4. Following the administration of RG barley chow, stool scores dropped below 0.4 also in
GS macaques.


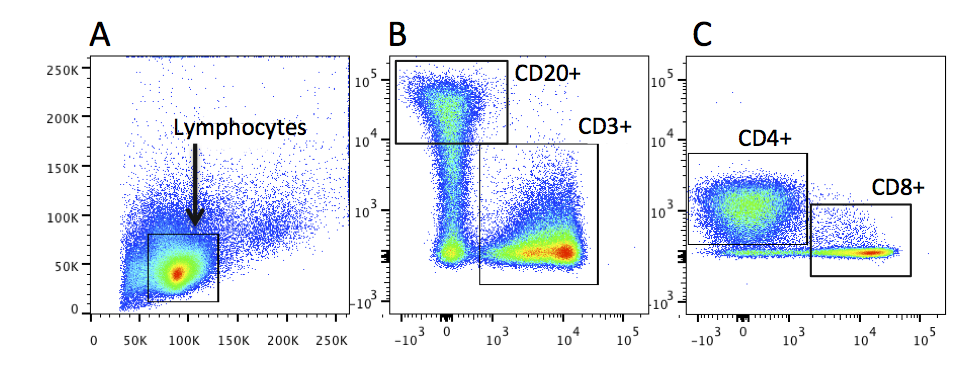


**Figure S2.** FACS gating strategy: The rhesus peripheral, mononuclear, lymphocyte-like cells (**A**) were gated on to identify the populations of CD3+ T and CD20+ B lymphocytes (**B**). In following step, populations of CD3+CD4+ T helper and CD3+CD8+ cytotoxic T cells were identified (**C**). The populations of CD3+CD4+, CD3+CD8+ and CD20+ lymphocytes were evaluated for the expression of selected pro- and anti-inflammatory cytokines. In addition, CD3+CD4+ T cells were evaluated for the expression of CD152 immunoregulatory molecule e.g., CTLA-4.


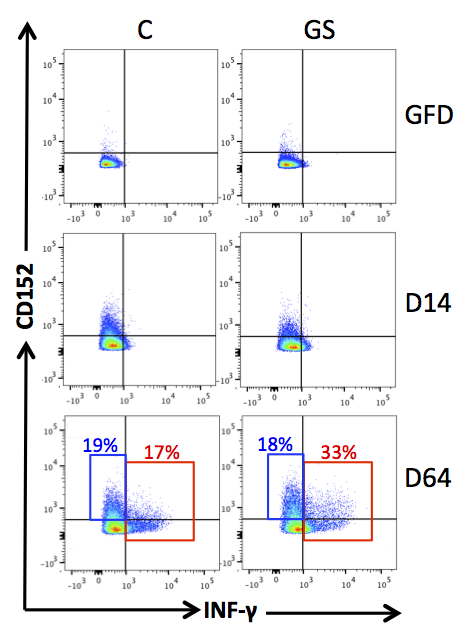


**Figure S3.** The proportion between the IFN-γ (marker of intestinal inflammation) and CD152 (marker of T cell inhibition) expression by peripheral CD3+CD4+ T cells was evaluated at different stages of barley gluten diet. The FACS histograms correspond to selected control (C) and GS macaques. While only the minimal expression of IFN-γ and CD152 was seen during immunological remission (GFD), introduction of barley diet led to an increased expression of both molecules by day 14 (D14) and further increase was observed by day 64 (D64). Total % of cells of interest is indicated by blue (CD3+CD4+CD152+) and red (CD3+CD4+IFN-γ+) squares.

© 2015 by the authors; licensee MDPI, Basel, Switzerland. This article is an open access article distributed under the terms and conditions of the Creative Commons Attribution license (http://creativecommons.org/licenses/by/4.0/).
